# Marijuana use among adolescents is associated with deleterious alterations in mature BDNF

**DOI:** 10.3934/publichealth.2019.1.4

**Published:** 2019-01-17

**Authors:** Maria Jose Miguez, Wenyaw Chan, Luis Espinoza, Ralph Tarter, Caroline Perez

**Affiliations:** 1 School of Integrated Science and Humanity, Florida International University, Miami, USA; 2 Department of Biostatistics and Data Science, University of Texas, Houston, USA; 3 Department of Medicine, University of Miami, Miami, USA; 4 Center for Education and Drug Abuse Research, University of Pittsburgh, Pittsburgh, USA

**Keywords:** Marijuana, adolescent, Brain Derived Neurotrophic Factor (BDNF), brain development, smoking

## Abstract

**Background:**

With increases in marijuana use and legalization efforts, it is imperative to establish its impact on the developing brain. Therefore, we investigated whether exposure to marijuana alters brain derived neurotropic-factor (BDNF), given its critical role in brain development and plasticity. We then examined whether onset age of cannabis use was associated with more severe changes. A single site, cohort study following 500 urban healthy American adolescents. Changes in plasma m-BDNF levels were longitudinally assessed, and a multi-method approach was implemented to ascertain marijuana use. Multivariate and general linear model (GLM) regression modeling were utilized to test the main hypothesis, controlling for confounders.

**Results:**

Group-based trajectory modeling identified four distinct groups, characterized by naive (60% control), starters (14%), chronic users (20%), and experimenting/quitters (6%). Compared to controls, those initiating marijuana use had similar pre-existent m-BDNF (1939.2 ± 221 vs. 2640.7 ± 1309 ng/ml, *p*=0.4) After adjusting for confounding factors, GLM analyses revealed that, compared to controls, younger adolescents increased BDNF levels when experimenting and during moderate marijuana use. Older adolescents had a steeper increase in endogenous BDNF levels, particularly when escalating use. Multivariate analyses confirmed marijuana use as a predictor of m-BDNF (p = 0.001).

**Conclusions:**

This is the first study demonstrating BDNF alterations were not a precondition. Rather, BDNF alteration was secondary to marijuana use, serving as cautionary evidence of marijuana's deleterious effects. Findings suggest that when marijuana use escalates, the BDNF pathway becomes more deregulated. Analyses confirm that age of marijuana use onset influences the magnitude of these changes.

## 1. Introduction

With approximately 147 million users, marijuana the cannabis sativa plant, is by far the most common drug used worldwide [1]–[2]. Noteworthy, global analyses revealed an overall rise in the prevalence of lifetime cannabis use by adolescents [3]. The dangers of marijuana products are disquieting when considering the increased potency of today's cannabis, and its rapid passage to a brain still under development [4].

Yet, the controversy over marijuana's dangers versus “safety” is ignited with contradictory findings. In addition, current knowledge is largely inferred from studies in animals, adults, and subjects with pathological conditions (i.e., schizophrenia) [5]–[8]. It is remarkable that very few studies focused on adolescents, even though the onset of marijuana use typically occurs during this developmental period. The ability to draw definitive conclusions from studies pertaining to the adolescent population is often limited by cross-sectional designs, the concurrent use of multiple substances, and the lack of information regarding pre-existing biological integrity [3],[9].

Adolescent exposure to cannabis normally occurs by smoking cannabis joints, which contain over 421 different chemicals, including over 60 cannabinoids, yet most in-vitro studies only use Δ9-tetrahydrocannabinol (Δ9-THC) at a fixed dose [10]. Active ingredients vary in concentration by strain (e.g., Sativa vs. Indicas), and their effects are not limited to the endocannabinoid system [11]. Moreover, they are the result of direct and indirect cellular and network effects. As a result of this faulty, simplistic approach, knowledge regarding marijuana's biological mechanisms of action is also limited.

Given that cannabinoids can transactivate BDNF receptors, and considering BDNF's critical role controlling brain development, cognitive processes, and neuroplasticity, it is surprising that very little research on BDNF in this context has been done [12]–[15]. Though BDNF has been widely studied for its ability to support neuronal development and plasticity, other less beneficial effects has been discovered. Data from animal models indicated, albeit with some exceptions, that BDNF also contributes to the enduring synaptic plasticity that underlies drug addiction [16]. The few studies examining the relationship between BDNF and marijuana in healthy humans has been inconclusive. They were mostly cross-sectional and performed on adults, and translating conclusions from them would be flawed, as marijuana leads to differential neurochemical effects in adolescence than during adulthood [6],[13],[17]. In addition, they used serum to measure BDNF, which is not considered a proxy of central nervous system levels, rather a proxy of platelets [9],[13]. Given all these methodological limitations, it is impossible to draw conclusions about the long-term effects of cannabis use on these at risk population [18]–[19].

Building on prior studies, our study aim was to determine if the BDNF pathway becomes deregulated with the use of marijuana. We followed the trajectories of adolescents before and after the onset of marijuana use. Our design considered prior methodological approaches and knowledge gaps. For example, unlike many previous studies, we used poor platelet plasma (PPP), as studies have consistently demonstrated that PPP is both significantly correlated with, and can be used as a proxy for, CNS levels [20]. We also carefully selected a healthy population with little to no use of other drugs to avoid their confounding effects.

## 2. Methods

“ROBIM” (the Role of Brain Derived Neurotrophic Factor in Decision Making Participants) is a 5-year, longitudinal study based in Miami, Florida. Adolescents were recruited through direct outreach to community and health care centers. Hispanic adolescents were eligible if they did not have a history of a clinical disease (i.e., cancer, renal or heart disease), major neurological, or psychiatric disorder (i.e., autism, severe developmental problems, schizophrenia) that prevented their participation in the study. Adolescents receiving any neuro-pharmacological intervention, or taking bodybuilding substances (i.e. steroids, growth hormones) were ineligible.

### 2.1. Data collection

Visits were conducted by trained interviewers and consisted of a detailed medical history, physical/neurological examinations, structured survey questionnaires, and a urine test to corroborate self-reported drug use. The protocol was approved by the IRB's at Florida International University and the University of Miami.

### 2.2. Cannabis measures

Using the NIDA Quick Screen, participants were queried regarding the use of marijuana at any point in their lives, as well as any other legal (alcohol or tobacco) or illegal drugs [20]. If the adolescent reported marijuana use, we asked after the intensity of use and age at first use. Intensity of use/the frequency of use was defined as once, once in the last 30 days, once a week, 3 times a week, 4 times a week, once daily, several times per day, and quit. The age of debut was included based on evidence suggesting that the greatest risk effects of cannabis occur during early adolescence, but are moderate to negligible when first used above the age of 18 [17].

On the basis of their answers over the length of the study, the participants were divided into categories/trajectories: no substance use, “once or twice” substance use [(likely no substance use disorder (SUD)], monthly use (likely mild-to-moderate SUD), weekly/daily (likely severe SUD) and those that quit. Each of these classifications corresponds to an “actionable category” as distinguished by AAP, which recommends a distinct type of brief intervention for each one [21].

### 2.3. Brain derived neurotrophic factor

BDNF is initially synthesized as a precursor (proBDNF), which is proteolytically processed into mature BDNF (mBDNF)[22]. Since BDNF cross the blood brain barrier, it can be measured in the periphery [23]. Blood was drawn between 8 and 11 a.m. to minimize the effects of circadian rhythm. Platelet-poor plasma (PPP) was obtained following standardized procedures and were assayed under blinded conditions. BDNF concentrations were quantitatively determined using the MILLIPLEX MAP Human Pituitary Magnetic Bead Panel from Millipore (EMD Millipore Corporation, Billerica, MA, USA). The assessments were done following the manufacturer's instructions.

### 2.4. Covariates

Information was collected on potentially confounding variables, including adolescent/parent sociodemographics (age, education, employment, gender, and country of birth) and medical history. We gathered information on variables known to affect BDNF, such as other abused drugs (e.g., amphetamines, barbiturates, tranquilizers, cocaine, heroin, opiates, PCP, psychedelics, inhalants, and steroids), exercise (Stanford 7-day survey), and body mass index [24].

### 2.5. Statistical analyses

Descriptive statistics (e.g., minimum, maximum, median, mean with SD for each variable, and the frequency and percentage for each categorical variable) were used to summarize the data. The dependent variable of interest was m-BDNF level.

A between-group analysis was adopted to compare the levels of BDNF between naïve, current, and past users of marijuana. For continuous demographic variables, a two-sample t test was used for comparing marijuana user and non-users groups. For categorical variables, a chisquare test was applied for this comparison.

Longitudinal linear mixed regression model was applied to examine the trend differences of BDNF between the two groups, separated by age younger than or older than 15 years old. Finally, multivariate regression analyses were also used to determine whether marijuana, contributed to the prediction of BDNF alterations, after sociodemographics, physical activity, nutrition, and other drugs were taken into account.

## 3. Results

### 3.1. Marijuana use

The proportion of adolescents reporting ever using marijuana at baseline was 26%, representing the most widely used substance in this population. An additional 5% reported trying marijuana in the past, but not using it anymore. By the 12-month evaluation, an additional 18 percent of the adolescents enrolled in the study began using marijuana. Use of other drugs (crack, cocaine, stimulants, prescription opiates, or club drugs) was only 5%.

The demographic characteristics of the adolescents by marijuana use groups (yes/no) are depicted in Table 1. The groups mostly differed in age; on average cannabis users were slightly older than non-users. Marijuana use was most prevalent among high school students (26%). Only 7% of middle school students reported current use of marijuana. As expected, at the beginning most cannabis use was intermittent, and low in amount, while older adolescents mostly reported weekly to daily use.

### 3.2. Mature BDNF in those beginning marijuana use

**Figure 1.**
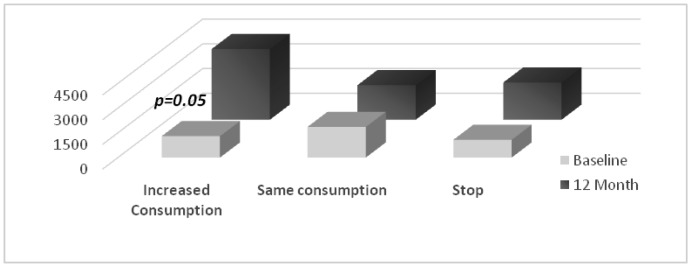
Impact of progressive marijuana use on BDNF levels. Pair wise comparisons of time points revealed that BDNF levels increased particularly in heavy users. BDNF remain high even after quitting suggesting that changes are long-lasting. Bars indicated the mean of the group at baseline (front line) and at the end of the study (back).

To resolve the quintessential dilemma of whether differences noted in BDNF levels can be attributed to predisposing characteristics, or whether they are a consequence of marijuana use, we evaluated BDNF levels in individuals that began using marijuana after baseline. By the 24-month visit, an additional 18% of the sample started using marijuana. Those initiating marijuana use have similar pre-existent m-BDNF levels compared to non-marijuana users (1939.2 ± 221 vs. 2640.7 ± 1309, *p* = 0.4). At baseline, m-BDNF levels were slightly lower in those progressing to higher consumption (1297.3 ± 278.7), than those who continued consuming at the same levels (1860.9 ± 176.2 pg/ml, *p* = 0.6). However, at the 12-month evaluation, m-BDNF values doubled for those with a pattern of escalating use (4284.5 ± 1942.3 vs. 2093.4 ± 292.1 pg/ml; *p* = 0.05 See Figure 1).

**Table 1.** Demographic characteristics of the adolescent population (n = °490).

Demographic Variable		Non Marijuana User	Marijuana User	*P* Values
Gender				
	Male	48%	45%	0.4
	Female	52%	55%	
Age In Years		14.5 ± 2.2	16.5 ± 1.4	< 0.001
Education		8.2 ± 2.3	10.0 ± 1.6	< 0.001
Income	Low/Poverty	41%	47%	
	Middle	26%	25%	0.4
	High Class	33%	28%	
Immigrant		23%	24%	
Born in the USA		77%	76%	0.9
Body Mass Index		23.3 ± 5.1	24.0 ± 6	0.5

Note: Differences in baseline sociodemographic measures between participants with and without marijuana use. Values are means ± SD or percentages. No significant differences in sociodemographic characteristics were reported between groups except for age and years of education.

### 3.3. Longitudinal analyses

As most features of addiction develop progressively as a consequence of repeated exposure to the specific drug, we analyzed the effect of marijuana use over the following 12 months. In contrast to baseline, high BDNF levels were evident among marijuana users compared to non-users (3731.1 ± 903.4 vs. 2046.2 ± 262.5, *p* = 0.02). In an effort to determine their trajectories, we compared changes longitudinally between younger adolescents <15 and those older.

Figures 2A and 2B illustrate the importance of considering developmental stage when analyzing BDNF levels. Figure 2A depicts the mean BDNF over time separated by marijuana user and non-users for those who started their marijuana at age ≤ 15. These models were based on longitudinal regression analysis, although marijuana use (p = 0.50), time (p = 0.24), and their interaction (p = 0.41) not significant. Figure 2B depicts the mean BDNF over time separated by marijuana users and non-users for those who started their marijuana at age > 15. These models were based on similar longitudinal regression analyses: marijuana use (p = 0.015), time (p < 0.0001), and their interaction (p = 0.012) were significant.

**Figure 2A.**
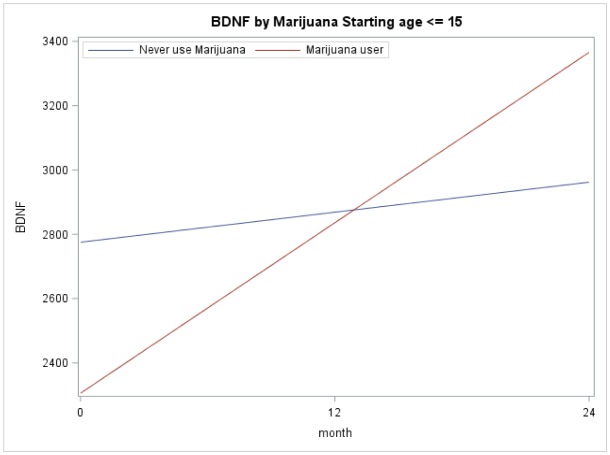
BDNF by Marijuana Starting Age ≤ 15. Illustrates the mean BDNF over time separated by marijuana user and non-users for those who started their marijuana use younger than 15.

**Figure 2B.**
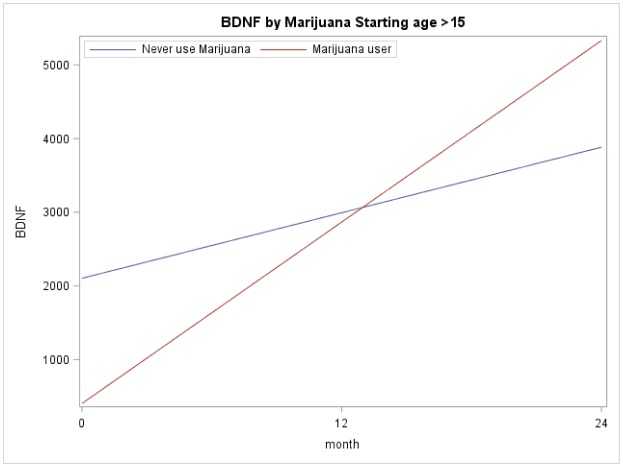
BDNF by Marijuana Starting Age > 15. Representation of m-BDNF levels at different time points and the trend of changes during the study period.

## 3.4. Final analyze

To assess whether marijuana use predicts BDNF levels, we performed a multivariate analysis using m-BDNF at the last visit as the dependent variable, and marijuana as the independent variable (see Table 2). To assure the absence of any other group differences, gender, age, body mass index, diet, exercise, stressors, and migration were included in the model as covariates. Significant effects for marijuana were found (*p*=0.014), meaning that marijuana use at baseline was associated with changes in BDNF levels.

**Table 2.** Regression analyses of BDNF at the last visit coefficients^a^.

Model	Unstandardized Coefficients		Standardized Coefficients	t	Sig.

	B	Std. Error	Beta		
(Constant)	3026.408	1752.859		1.727	0.091
Marijuana use	274.89	136.97	0.120	2.007	0.04
Age	−151.394	111.915	−0.083	−1.912	0.062
Migration/stress	440.621	581.792	0.046	3.082	0.449
Body Mass Index	−2.976	46.966	−0.04	−0.063	0.950
Gender	−558.545	478.830	−0.069	−1.166	0.244

Note: P value using Logistic Regression likelihood ratio adjusted for sociodemographic, migration/stressors, family (income, attachment, type of family) and healthy behavior variables (nutrition, exercise, other drugs). Age and marijuana use were the only predictors of BDNF level at the last visit.

## 4. Discussion

The Healthy People 2020 initiative has set the goal to reduce the rate of adolescent marijuana use to six percent [25]. However, it only takes one look at the data from our sample (25% are regular users) to recognize that we are far from that goal. This widespread use should be alarming, since adolescence is a peak time for neurodevelopment, and subsequently a period of increased susceptibility to alterations in brain structure and function [2],[6]–[8],[26]–[29]. The present study specifically provides evidence that both initiation and regular use of marijuana predict changes in mature BDNF.

Although it is inherently difficult to establish causality in human studies, our methodological and design approaches provide a stronger basis for causal inference [30]. First, this study was longitudinal, which allowed for uniformity. Secondly, by including a sizable proportion of drug naïve subjects, and by performing drug screening tests, we were able to establish that drug use preceded the outcome. Our study took measures to exclude alternative explanations for our findings. Participants passed a rigorous health screening, including medical history, physical exam, and blood testing to assure the inclusion of adolescents without comorbid developmental or neurologic conditions. We also collected information on several covariates that potentially impact BDNF, such as diet, exercise, sociodemographics, and other drug use. Finally, animal models have long provided support that the observed relationship between marijuana and BDNF is more than a simple association [4]–[5],[12]–[13],[15],[28]–[29]. They have also provided the mechanisms of action by which these variables interact: a) activation of the extracellular signal-regulated kinase or b) through endocannabinoid transactivation of BDNF-TrkB receptors [16],[31].

While the increased levels of m-BDNF associated with cannabinoids have been previously reported, the functional relevance of these changes should be carefully interpreted [6],[13]. Though in the past they were viewed as beneficial, this is an erroneous deduction [6],[13]. This conclusion is derived from acute models where increases in m-BDNF protect the brain in the short-term [32]. Subsequent research has established that chronic exposure will lead to allostatic overload, maladaptive responses, and illness [33]. Additionally, a rise in BDNF, which is highly distributed in the hippocampus, increases the odds of hyperexcitability and/or excitotoxic damage by increasing long term potentiation [34]. Further confirming our postulates are human neuroimaging studies showing that marijuana use is associated with a volumetric reduction of the hippocampus [31].

By modifying BDNF, marijuana use could also induce alterations in “plasticity”, which reference neuronal changes associated with the acquisition of new skills or the ability to adapt [35]–[37]. Emerging data have demonstrated that synaptic plasticity may also be involved in the “learning” of addictive behaviors [16],[38]–[40]. In vertebrate model systems, exogenous BDNF promotes cocaine-taking behavior over a range of experimental conditions, and led to increased risk of relapse [41]. Moreover, BDNF infusions induced a switch in the γ-aminobutyric acid type A receptors from inhibitory to excitatory signaling, potentiating cue-induced drug seeking [39]. These findings from different fields let us conclude that sustained increases of m-BDNF are not beneficial. Translational studies are needed to confirm whether this interaction reflects a drug-induced, synaptic plasticity phenomenon similar to the one observed in animals [16],[39]–[41].

In addition, our data demonstrated that there are variations in BDNF responses between younger and older individuals. Since marijuana use among the younger teens in our study was generally “light”, it is possible to conclude that a dose-response should exist. The steep increases in BDNF associated with escalating marijuana use also suggested this trend. This phenomenon may result from BDNF-induced neuroplasticity changes that promote drug seeking [38]. Such activity-dependent remodeling has been proposed as part of the transition from casual drug use to drug addiction in other drug fields [39]–[40]. Supporting these posits are also data demonstrating that injection of a TrkB antagonist in the peripheral system significantly reduces drug-dependency behaviors in animals [40]–[42].

In summary, marijuana the most frequent drug used by these adolescents alter the production of BDNF. Alterations were both age and dose dependent and while past users improved their levels they did not return to basal levels. Analyses confirmed that they were not a pre-condition that leads to increase substance use. Our next goal is to analyze the consequences in neuropsychological performance to better understand the neurodevelopmental trajectories of users versus non-users.

These relevant findings come with some caveats. Our sample was limited to Hispanic adolescents in South Florida. Nevertheless, our study was longitudinal and had a good sample size for a single site study. BDNF levels were measured in plasma and not in the CNS. However, both in humans and in animals, peripheral levels highly correlated with changes observed in the brain [20],[43]–[44]. Though it is inherently difficult to establish causality in human studies, we ensured that conclusions were determined with a high level of confidence by using the correct design and laboratory methods. Animal models also provide strong support that the observed relationship between marijuana and BDNF is more than a simple association [5]–[6],[10]–[11],[16],[29]–[30]. They also provide support that such alterations could be deleterious during critical developmental periods, and could contribute to, and exacerbate, addictive behavior. Although, marijuana users exhibited lower BDNF levels raising a question about their potential predicting value, additional studies should be performed in order to confirm our findings. Finally, results indicated the importance of analyzing marijuana across the different phases of adolescence, as it can be more informative when determining long-term impact.

Now policy initiatives need to ensure that legalization of marijuana to protect the rights of ailing individuals does not harm the youth who will be the future of every country. It is clear from the data amassed herein that the adolescent body registers marijuana use as a stressor. Effects of chronic use remain pervasive via heightened m-BDNF, leading to alterations in brain function and structure [6],[26]–[29]. Such results caution against movements to broadly legalize marijuana.
